# Monophonic rubbling number of some standard graphs

**DOI:** 10.1016/j.heliyon.2024.e31679

**Published:** 2024-05-22

**Authors:** K.C. Kavitha, S. Jagatheswari

**Affiliations:** Department of Mathematics, School of Advanced Sciences, Vellore Institute of Technology, Vellore, Tamil Nadu, India

**Keywords:** Rubbling number, Monophonic rubbling number

## Abstract

Let G be a connected graph with vertices V and edges E. Rubbling is a recent development in graph theory and combinatorics. In graph rubbling an extra shift is allowed that adds a pebble at a node after the deletion of one pebble each at two neighbouring vertices. For the first time, we introduce the concept of monophonic rubbling numbers into the literature. A monophonic rubbling number, $\mu_{r}(G)$, is the least number *m* required to ensure that any vertex is reachable from any pebble placement of *m* pebbles using a monophonic path by a sequence of rubbling shifts. We calculate the upper bound and lower bound using the monophonic rubbling number of some standard graphs and derived graphs.

## 1 Introduction

We have considered a connected graph G with the vertex set V(G) and edge set E(G). Graph pebbling is a concept in graph theory and combinatorics. It is a model used for the transportation of resources. We can consider pebbles as a fuel container. Then the loss of pebbles will be the cost of transportation. Lagarias and Saks introduced graph pebbling and it was introduced into the literature by Chung [1]. Initially, pebbles are distributed on the vertices on graph G such that each vertex of V is assigned a positive integer number of pebbles called the pebble distribution on graph G. The pebbling shift is the deletion of two pebbles from a vertex and placing one pebble to a neighbouring vertex. The pebbling number of a graph G is defined as f(G), which is the minimum number *k* in which, for any distribution of *k* pebbles on the vertices of a graph G, we can move a pebble to any of the specific vertex by a sequence of pebbling shifts. In [2] Glenn Hurlbert studied an overview of graph pebbling number.

C. Belford and N. Sieben introduced rubbling number in [3]. Rubbling is a version of graph pebbling with an extra shift. An additional shift is the only difference between a pebbling number and rubbling number. In the rubbling concept, two vertices adjacent to a specified vertex have one pebble each, and then from those two vertices, we can shift a pebble to the specified vertex. The vertex v in graph G is reachable if a pebble can be placed on v using a sequence of pebbling and rubbling shifts. In graph pebbling only pebbling shift is permitted whereas in rubbling both pebbling and rubbling shift is permitted [4]. C. Belford et al. in [3] computed the rubbling number and optimal rubbling number in some standard graphs. GY Katona et al. computed the upper bound for the rubbling of n-vertex, and diameter d-graphs and estimated the maximum rubbling number of diameter 2 graphs in [5]. GY. Katona et al. computed the optimal rubbling number of the ladder graph, prism graph and Mobius ladder graph in [6]. Nanbor Seiben briefly discussed graph pebbling number and graph rubbling number in [7]. Zheng Jiang Xia et al. computed the rubbling number on the cycle graph and optimal rubbling number of paths, cycles, and grid on $P_{2}\times P_{n}$ graphs and upper bound of the $P_{m}\times P_{n}$ graph in [8]. Laszlo F. Papp et al. computed the lower bounds on both the optimal pebbling and rubbling number using the distance k domination number in [9]. Robert A. Beeler et al. computed the domination cover rubbling number of standard graphs such as path, cycle, and trees and found their bounds in [4]. In article [10] the author studied $C_{8}$-super magic labelling of zig-zag, linear chains and also the disjoint union of non-isomorphic copies of both chains which motivated us to implement the cycle graph $C_{m}$ into our study.

A monophonic path between two vertices say a and b is the a-b path which is chordless. A chord is the line segment that joins any two points a and b. Dhivviyanandam et al. introduced the monophonic pebbling number, monophonic t-pebbling number in [11][12][13][14][15] and computed the same for several graphs. For the first time in the literature, we introduce the monophonic rubbling number concept. Here, we study the monophonic rubbling number, $\mu_{r}(G)$, of path graph, cycle graph, complete graph, wheel graph, n-star graph, complete bipartite graph, fan graph, ladder graph, middle of a path graph and square of paths.

## 2 Motivation

We discuss the limitations mentioned in the introduction which in turn motivated this study:

• The monophonic pebbling number related articles in [11][12][13][14][15] motivated to explore the new topic on monophonic rubbling number.
• The number of pebbles required is more using monophonic pebbling number. This motivated us to introduce monophonic rubbling number into the literature, which provides the minimum number of pebbles with an additional move.
• Traditional methods of pebbling concepts like cover rubbling number [16], total domination cover rubbling number [17] is time-consuming and require more pebbles.

## 3 Graph preliminaries

Definition 3.1 A monophonic rubbling number, $\mu_{r}(G,v)$, is the least number *m* required to ensure that any vertex is reachable from any pebble distribution of *m* pebbles using a monophonic path by a sequence of rubbing shifts. A monophonic path between any two points is a chordless path. The highest, $\mu_{r}(G,v)$, compared to all the vertices of G is the monophonic rubbling number of a graph, denoted as $\mu_{r}(G,v)$.

Definition 3.2 [18] Assume the path $P_{k-1}$: $x_{1},\cdots ,x_{k-1}$. On attaching an extra vertex $x_{0}$ to the path $P_{k-1}$ and join it to all of the path vertices. The graph formed from this process is called a fan graph.

Definition 3.3 [6] A Ladder graph ($L_{k}$) is a cartesian product of $P_{k}$ = $P_{2}\times P_{k}$ where $P_{k}$ is a path graph with k-vertices. The ladder graph has 2k vertices and 3k - 2 edges.

Definition 3.4 [19] A middle graph ($M_{k}$) in a path graph $P_{k}$ is obtained by inserting a vertex between every pair of vertices in a path graph $P_{k}$.

Definition 3.5 If there are $\left\{v_{1},v_{2},\cdots ,v_{k}\right\}$ vertices, the square of path $P_{k}^{2}$ is formed by joining the edges as $\left(v_{i}v_{i+1}),(v_{j}v_{j+1}\right)$ where i = {1,2,3,⋯,k−1} and j = {1,2,3⋯,k−2}.

Definition 3.6 [11] For a given graph G, a transmitting subgraph of G is a path $v_{0},v_{1},v_{2},\cdots ,v_{k}$ in which there are at least 2 pebbles on $v_{0}$ and one pebble each on the other vertices except the target vertex such that we can move a pebble from $v_{0}$ to $v_{k}$.

Theorem 3.1 *[3]* *The rubbling number of the path graph with k-vertices is* $2^{k-1}$ *.*

## 4 Remark

• If the vertices adjacent to the target vertex x are y and z which has a pebble each, then we can shift a pebble to the target vertex from those two vertices using rubbling shifts.
• Among the two adjacent vertices y and z, if any vertex has 2 pebbles and the other has a pebble, then we can shift a pebble to the target vertex x using rubbling shifts.
• Let *γ* be the destination vertex.
• Let μ(G) be the monophonic pebbling number.

## 5 The algorithm for finding the monophonic rubbling number for any given graph G(V, E)

Here the generalized algorithm for finding the monophonic rubbling number is introduced.

**Step 1**. Firstly, the destination to be fixed.

**Step 2**. For a given graph G(V, E), the monophonic distance $d_{M}$ needs to be calculated for all vertices.

**Step 3**. Compare the monophonic distances of all the nodes. Among which choose the maximum monophonic length $d_{M}$ which is chordless.

**Step 4**. The monophonic path(P) needs to be fixed based on the longest monophonic distance $d_{M}$.

**Step 5**. Find the monophonic rubbling number for each node considering the pebbling move along the monophonic path which is chordless. Along with the pebbling move, an additional move is also considered i.e., when two vertices say a and b adjacent to the target vertex have a pebble each, then using rubbling move we can transfer a pebble to the target vertex.

**Step 6**. The node which has the least upper bound number of pebbles will be the monophonic rubbling number of the graph G(v, E).

**Step 7**. Let us assume the monophonic path $\left(P_{1}\right)$ which has the maximum monophonic distance $d_{M}$.

**Step 8**. Placing some pebbles on the nodes which are not on the monophonic path $\left(P_{1}\right)$ in such a way that no pebbles will reach the path $\left(P_{1}\right)$.

**Step 9**. Let us denote the nodes which are not on the monophonic path $P_{1}$ as $P_{1}^{\text{‘}}$. Now based on the monophonic distance $d_{M}$ we get $2^{d_{M}}$.

**Step 10**. Now adding pebbles on the nodes of the monophonic path and pebbles on the nodes which are not on the monophonic path.

Hence, $\mu_{r}(G(v,E))$= $2^{d_{M}}+p(V(P_{1}^{\text{‘}}))$.

**Step 11**. This is the least upper-bound monophonic rubbling number when we compare it with the rest of the nodes. Thus, we arrive at a monophonic rubbling number of any graph for all the distributions.

## 6 Main results

Theorem 6.1 *For any path graph* $\left(P_{k}\right)$ *, the monophonic rubbling number is* $\mu_{r}(P_{k})$ *=* $2^{k-1}$ *.*

Proof Consider the vertex set of $P_{k}$ be written as $V(P_{k})$ = $\left\{v_{1},v_{2},v_{3},\cdots ,v_{k}\right\}$. Let *γ* = $v_{1}$. If $p(v_{k})$ = $2^{k-1}-1$, a pebble cannot be moved to *γ*. Hence, $\mu_{r}(P_{k})$ ≥ $2^{k-1}$.

To prove for sufficient condition, let D be any scattering of $2^{k-1}$ pebbles on the vertices of $P_{k}$.

**Case 1:** Let *γ* be any corner vertices.

Without loss of generality, let *γ* = $v_{k}$. The monophonic length from $v_{k}$ to any other vertex in $P_{k}$ is k - 1. We can transfer one pebble to the destination *γ* by scattering $2^{k-1}$ pebbles on the vertices of the path graph $P_{k}$ by Theorem 3.1.

**Case 2:** Let *γ* = $v_{m}$ where m = {2,3,⋯,(k−1)}.

The monophonic length from $v_{m}$ to $v_{i}$ is atmost (k - m), where m < i ≤ k. By Theorem 3.1., if $p(<P_{k}-\{v_{m},v_{m+1},\cdots ,v_{k}\}>)$ ≥ $2^{k-m}$ pebbles, we can transfer one pebble to the target. Otherwise, we can shift a pebble to the target if $<v_{1},v_{2},\cdots ,v_{m}>$ contains at least $2^{m-1}$ pebbles. Now $2^{m-1}$ pebbles is enough to reach the target since the monophonic length from $v_{m}$ to $v_{t}$ is at most m - 1, where 1 ≤ t < m. Therefore, the monophonic rubbling number for the path graph $P_{k}$ is $\mu_{r}(P_{k})$ = $2^{k-1}$. Hence the proof.

Theorem 6.2 *For any cycle graph (* $C_{k}$ *), the monophonic rubbling number is* $\mu_{r}(C_{k})$ *=* $2^{k-2}$ *.*

Proof Consider the vertex set of cycle graph $\left(C_{k}\right)$ be written as $V(C_{k})$ = $\left\{v_{1},v_{2},\cdots ,v_{k}\right\}$. Let *γ* = $v_{1}$. On placing $2^{k-2}$ - 1 pebbles on the vertex $v_{k-1}$, the destination is not reached using the monophonic path. Therefore, the monophonic rubbling number is $\mu_{r}(C_{k})$ ≥ $2^{k-2}$.

Let D be any scattering of $2^{k-2}$ pebbles on the vertices of $C_{k}$ to prove the sufficiency condition.

Let *γ* be any vertex of the cycle $C_{k}$.

Without loss of generality, let *γ* = $v_{k-1}$. The monophonic length from $v_{k-1}$ to any vertex in $C_{k}$ is (k - 2). Assume the monophonic path P: $v_{1}\to v_{2}\to \cdots ,\to v_{k-1}$. By distributing $2^{k-2}$ pebbles on the path P, we can transfer a pebble to the destination vertex *γ*. By scattering $2^{k-2}$ - 1 pebble on the path P and placing one pebble on any one of the vertices except *γ*, using rubbling move we can reach the destination. Similarly, we can prove for $v_{j}$ where j = {1,2,⋯,k−2,k}. Therefore, the monophonic rubbling number for the cycle graph is $\mu_{r}(C_{k})$ = $2^{k-2}$. Hence the proof.

Theorem 6.3 *For any complement of cycle graph (* ${\bar{C}}_{k}$ *) for k >4, the monophonic rubbling number is* $\mu_{r}({\bar{C}}_{k})$ *= 8.*

Proof Consider the vertex set of the complement of cycle graph ${\bar{C}}_{k}$ be written as $V({\bar{C}}_{k}$) = $\left\{v_{1},v_{2},v_{3},\cdots ,v_{k}\right\}$. Let *γ* = $v_{1}$. Consider the monophonic path P: $v_{2}\to v_{k}\to v_{4}\to v_{1}$. On placing 7 pebbles on path P, the destination is not reached. Therefore, the monophonic rubbling number of the complement of cycle graph ${\bar{C}}_{k}$ is $\mu_{r}({\bar{C}}_{k})\geq 8$.

To prove the sufficiency condition, let D be any scattering of 8 pebbles on the vertices of the complement of the cycle graph.

Let *γ* = $v_{k}$. Consider the monophonic path $P_{1}$: $v_{k-1}\to v_{2}\to v_{k-2}\to v_{k}$. The monophonic length from *γ* to any other vertex is 3. On placing 8 pebbles on the path, the destination is reached. On placing 7 pebbles on the path $P_{1}$ and placing a pebble on any one of the vertices, by taking an alternative path of the same distance, the destination is reached. On distributing one pebble each on any of the 8 vertices, we can reach the destination using rubbling move. By placing 2 pebbles on the vertex adjacent to the target vertex, the destination is reached. By symmetry, we can prove for other vertices. Hence, for all the vertices of the complement of cycle graph ${\bar{C}}_{k}$, the destination is reached. Therefore, the monophonic rubbling number of the complement of cycle graph ${\bar{C}}_{k}$ is $\mu_{r}({\bar{C}}_{k})$ = 8. Hence the proof.

Theorem 6.4 *For any complete graph (* $K_{k}$ *), the monophonic rubbling number is* $\mu_{r}(K_{k})$ *= 2.*

Proof Let the vertex set of $K_{k}$ be written as $V(K_{k})$ = $\left\{v_{1},v_{2},\cdots ,v_{k}\right\}$. Let *γ* = $v_{1}$. The monophonic distance from *γ* to any other vertex is 1. Using a pebble we cannot reach the destination *γ*. Therefore, the monophonic rubbling number is, $\mu_{r}(K_{k})$ ≥ 2. Since in a complete graph, every vertex is connected with every other vertex, with 2 pebbles, the destination is reached. Therefore, the monophonic rubbling number of complete graph $K_{k}$ is $\mu_{r}(K_{k})$ = 2. Hence the proof.

Theorem 6.5 *For any wheel graph (* $W_{k}$ *), the monophonic rubbling number is* $\mu_{r}(W_{k})$ *=* $2^{k-3}$ *.*

Proof Let the vertex set of $W_{k}$ be written as $V(W_{k})$ = $\left\{v_{1},v_{2},\cdots ,v_{k}\right\}$ where $v_{1}$ be the centre vertex. Let *γ* = $v_{2}$. Consider the monophonic path P: $v_{k-1}\to v_{k-2}\to ,\cdots ,\to v_{3}\to v_{2}$. The vertices $v_{1}$ and $v_{k}$ are non-monophonic vertices. On placing $2^{k-3}-1$ pebbles on the path P, the destination is not reached using the monophonic path. Therefore, the monophonic rubbling number is, $\mu_{r}(W_{k})$ ≥ $2^{k-3}$.

To prove for sufficient condition, let D be any scattering of $2^{k-3}$ pebbles on the vertices of the graph $\left(W_{k}\right)$.

**Case 1:** Let *γ* = $v_{1}$.

Consider $p(v_{1})$ = 0. Assume the monophonic path $P_{1}$: $v_{k}\to v_{1}$. The monophonic length from *γ* to any other vertex is 1. Since $v_{1}$ is connected to all other vertices, using 2 pebbles the destination *γ* is reached.

**Case 2:** Let *γ* = $v_{k-1}$.

Assume the monophonic path $P_{2}$: $v_{2}\to v_{3}\to ,\cdots ,\to v_{k-1}$. The monophonic length from *γ* to any other vertex is (k - 3). On placing $2^{k-3}$ pebbles on the path $P_{2}$, the destination is reached. Similarly, we can prove this for other vertices. Therefore, the monophonic rubbling number of wheel graph $W_{k}$ is $\mu_{r}(W_{k})$ = $2^{k-3}$. Hence the proof.

Theorem 6.6 *For any k-star graph (* $S_{1,k}$ *), the monophonic rubbling number of n-star graph* $S_{1,k}$ *is* $\mu_{r}(S_{1,k})$ *= 4.*

Proof Consider the vertex set of $S_{1,k}$ be written as $V(S_{1,k})$ = $\left\{u_{1},v_{1},v_{2},\cdots ,v_{k}\right\}$ where $\left\{v_{1},v_{2},\cdots ,v_{k}\right\}$ are the pendent vertices and $u_{1}$ is the middle vertex. Let *γ* = $v_{1}$. By placing 3 pebbles on the vertex $v_{k}$, the destination is not reached. Therefore, the monophonic rubbling number of n-star graph ($S_{1,k}$) is $\mu_{r}(S_{1,k})$ ≥ 4.

To prove for sufficiency condition, let D be any scattering of 4 pebbles on the vertices of the n-star graph.

**Case 1:** Let $v_{k}$ be the destination vertex.

On placing 4 pebbles on any one of the vertices, the destination is reached. On placing 2 pebbles on any two vertices, by bringing a pebble to the centre vertex $u_{1}$, the destination is reached. On placing one pebble each on any 4 vertices, the destination is reached. On placing 3 pebbles on any one of the vertices and placing a pebble on any one of the remaining vertices, the destination is reached.

**Case 2:** Let $u_{1}$ be the destination vertex.

On placing 2 pebbles on any one of the vertices except the target vertex, the destination is reached. On placing one pebble each on two vertices, using rubbling move, the destination is reached. Hence, $\mu_{r}(S_{1,k})$ ≤ 4. Thus, $\mu_{r}(S_{1,k})$ = 4. Hence the proof.

Theorem 6.7 *For any complete bipartite graph (* $K_{m,n}$ *), the monophonic rubbling number is* $\mu_{r}(K_{m,n})$ *= 4.*

Proof Consider the vertex set of $K_{m,n}$ be written as $V(K_{m,n})$ = $\left\{v_{1},v_{2},\cdots ,v_{m},u_{1},u_{2},\cdots ,u_{n}\right\}$. Let *γ* = $v_{1}$. Consider the monophonic path P: $v_{m}\to u_{1}\to v_{1}$. The other vertices are non-monophonic. Using 3 pebbles on the path P, the destination *γ* is not reached. Therefore, the monophonic rubbling number is $\mu_{r}(K_{m,n})$ ≥ 4.

To prove for sufficiency condition, let D be any scattering of 4 pebbles on the vertices of the graph $K_{m,n}$. Let *γ* = $v_{m}$. Consider the monophonic path $P_{1}$: $v_{1}\to u_{1}\to v_{m}$. The monophonic length from $v_{m}$ to any other vertex is 2. On placing 4 pebbles on the path $P_{1}$, the destination is reached. Therefore, the monophonic rubbling number of complete bipartite graph $K_{m,n}$ is $\mu_{r}(K_{m,n})$ = 4. Hence the proof.

Theorem 6.8 *For any fan graph (* $F_{k}$ *), the monophonic rubbling number is* $\mu_{r}(F_{k})$ *=* $2^{k-2}$ *.*

Proof The fan graph is constructed as $F_{k}$ = $K_{1}+P_{k-1}$. Consider the vertex set of $K_{1}$ be written as $V(k_{1})$ = $v_{1}$. Let $V(P_{k-1})$ = $\left\{x_{1},x_{2},\cdots ,x_{k-1}\right\}$. In total, the vertices of the set $F_{k}$ = $\left\{v_{1},x_{1},x_{2},\cdots ,x_{k-1}\right\}$. Let *γ* = $x_{1}$. Consider the monophonic path P: $x_{k-1}\to x_{k-2},\cdots ,\to x_{2}\to x_{1}$. The monophonic length from *γ* to any other vertex is (k - 2). The vertex $v_{1}$ is not on the monophonic path. On placing $2^{k-2}-1$ pebbles on the path P, the destination is not reached. Therefore, the monophonic rubbling number is $\mu_{r}(F_{k})$ ≥ $2^{k-2}$.

To prove the sufficiency condition, let D be any scattering of $2^{k-2}$ pebbles on the vertices of the fan graph $F_{k}$.

**Case 1:** Let *γ* = $v_{1}$.

Consider the monophonic path $P_{1}$: $v_{1}\to x_{1}$. The monophonic length from *γ* to any other vertex is 1. On placing 2 pebbles on the path $P_{1}$, the destination is reached. On placing a pebble each on any two vertices, using rubbling moves, the destination is reached.

**Case 2:** Let *γ* = $x_{1}$.

Assume the monophonic path $P_{2}$: $x_{k-1}\to x_{k-2},\to ,x_{2},\to x_{1}$. The monophonic length from *γ* to any other vertex is (k - 2). If pebbles on the path $P_{2}$ have $2^{k-2}$ pebbles, the destination is reached. On placing one pebble on the vertex $v_{1}$ and placing $2^{k-2}-1$ pebbles on the path $P_{2}$, the destination is reached. By symmetry, we can prove for other vertices of the graph. Therefore, the monophonic rubbling number of fan graph $F_{k}$ is $\mu_{r}(F_{k})$ = $2^{k-2}$. Hence the proof.

**Case 3:** Let *γ* = $x_{m}$ where m = {2,3,⋯,(k−1)}.

The monophonic distance from $x_{m}$ to $x_{i}$ is atmost (k - m), where m < i ≤ k. By Theorem 3.1, if $p(<F_{k}-\{x_{m},x_{m+1},\cdots ,x_{k}\}>)$ ≥ $2^{k-m}$ pebbles, we can transfer a pebble to the target. Otherwise, we can shift a pebble to the target if $<x_{1},x_{2},\cdots ,x_{m}>$ contains at least $2^{m-1}$ pebbles. Now $2^{m-1}$ pebbles are enough to reach the target since the monophonic length from $v_{m}$ to $v_{t}$ is at most m - 1, where 1 ≤ t < m. Therefore, the monophonic rubbling number for the fan graph $F_{k}$ is $\mu_{r}(F_{k})$ = $2^{k-2}$. Hence the proof.

Theorem 6.9 *For any ladder graph (* $L_{k}$ *), the monophonic rubbling number is* $\mu_{r}(L_{k})$ *=* $2^{k+2}$ *.*

Proof Let the vertex set of the ladder graph ($L_{k})$ be written as $V(L_{k})$ = $\left\{v_{1},v_{2},\cdots ,v_{2k}\right\}$. Let *γ* = $v_{1}$. Consider the monophonic path P: $v_{1}\to v_{2},\cdots ,v_{k-1}\to v_{2k-1}\to v_{2k}$. On placing $2^{k+2}-1$ pebbles on the path P, the destination is not reached. Therefore, the monophonic rubbling number of ladder graph $L_{k}$ is $\mu_{r}(L_{k})$ ≥ $2^{k+2}$.

To prove the sufficiency condition, let D be any scattering of $2^{k+2}$ pebbles on the vertices of the ladder graph $L_{k}$.

**Case 1:** Let *γ* be any corner vertex.

Consider the monophonic path $P_{1}$: $v_{1}\to v_{3}\to v_{5},\cdots ,\to v_{2k-1}\to v_{2k}$. The monophonic length from *γ* to any other vertex is (k + 2). On placing $2^{k+2}$ pebbles on the path $P_{1}$, the destination is reached. On placing $2^{k+2}-1$ pebbles on the path $P_{1}$ and placing a pebble on any one of the remaining vertices, the destination is reached.

**Case 2:** Let *γ* = $v_{d}$ where d = {3,4,⋯,(2k−3),(2k−2)}.

The monophonic distance from $v_{d}$ to $v_{i}$ is atmost k, where d < i ≤ 2k. By Theorem 3.1., if $p(<L_{k}-\{v_{d},v_{d+1},\cdots ,v_{2k}\}>)\geq 2^{k}$ pebbles, the destination is reached. Otherwise, we can move a pebble to the target if $<v_{1},v_{2},\cdots ,v_{k}>$ contains at least $2^{d-1}$ pebbles. Now $2^{d-1}$ pebbles are enough to reach the target since the monophonic length from $v_{d}$ to $v_{t}$ is at most d−1, where 1 < t < d. Therefore, the monophonic rubbling number of the ladder graph ($L_{k}$) is $\mu_{r}(L_{k})$ = $2^{k+2}$. Hence the proof.

Theorem 6.10 *For every middle graph in a path graph* $M(P_{k})$ *with 2k - 1 vertices, the monophonic rubbling number is* $\mu_{r}(M(P_{k}))$ *=* $2^{2k-2}$ *.*

Proof Let the vertex set of the path graph $P_{k}$ be written as $\left\{v_{1},v_{2},\cdots ,v_{k}\right\}$ and the vertex set of the inserted graph be written as $\left\{u_{1},u_{2},\cdots ,u_{k-1}\right\}$. Let *γ* = $v_{1}$. On placing $2^{2k-2}-1$ pebbles on the vertex $v_{k}$, the destination is not reached. Therefore, the monophonic rubbling number is, $\mu_{r}(M(P_{k}))$ ≥ $2^{2k-2}$.

To prove for sufficiency condition, let D be any scattering of $2^{2k-2}$ pebbles on the vertices of the middle graph $M(P_{k})$.

**Case 1:** Let *γ* be any corner vertex. Let *γ* = $v_{k}$.

Consider the monophonic path $P_{1}$: $v_{1}\to v_{2}\to ,\cdots ,\to v_{k}$. The monophonic length from *γ* to any other vertex is (2k - 2). From Theorem 6.1., we can reach the destination. Similarly, we can prove for the vertex $v_{1}$. Now let *γ* = $v_{i}$ where 1 <i <k. Consider the monophonic path $P_{2}$: $v_{i}\to u_{i}\to v_{i+1}\to u_{i+1},\cdots ,\to v_{k}$. The monophonic length from *γ* to any other vertex is (2k - 2i). On placing $2^{2k-2i}$ pebbles on the path $P_{2}$, the destination is reached. Otherwise, consider the monophonic path $P_{3}$: $v_{1}\to u_{1}\to ,\cdots ,\to v_{i}$. The monophonic length from *γ* to any other vertex is (2i - 2). On placing $2^{2i-2}$ pebbles on the path $P_{3}$, the destination is reached. So, $p(P_{2}+P_{3})$ = $2^{2k-2i}$ + $2^{2i-2}$ = $2^{2k-2}$.

**Case 2:** Let *γ* = $u_{i}$ where i = 1 <i <k - 1.

Assume the monophonic path $P_{4}$: $u_{i}\to v_{i+1}\to u_{i+1}\to ,\cdots ,\to v_{k}$. The monophonic length from *γ* to any other vertex is 2k - 2i - 1. On placing $2^{2k-2i-1}$ pebbles on the path $P_{4}$, the destination is reached. Otherwise, assume the monophonic path $P_{5}$: $u_{i}$ to $u_{1}$. The monophonic distance from *γ* to any other vertex is 2i - 1. On placing $2^{2i-1}$ pebbles on the path $P_{5}$, the destination is reached. So, $p(P_{4}+P_{5})$ = $2^{2k-2i-1}$ + $2^{2i-1}$ = $2^{2k-2}$. Therefore, the monophonic rubbling number of middle graph $M(P_{K})$ is $\mu_{r}(M(P_{k}))$ = $2^{k-2}$. Hence the proof.

Theorem 6.11 *For any square of paths* $P_{k}^{2}$ *, the monophonic rubbling number is* $\mu_{r}(P_{k}^{2})$ *=* $2^{k-(\lceil \frac{k}{3}\rceil +1)}$ *.*

Proof Let the vertex set of the square of paths ($P_{k}^{2}$) be written as $V(P_{k}^{2})$ = $\left\{v_{1},v_{2},\cdots ,v_{k}\right\}$ and edge set be written as $E(P_{k}^{2})$ = $\left(v_{i}v_{i+1}),(v_{j}v_{j+1}\right)$ where i = {1,2,⋯,k−1} and j = {1,2,⋯,k−2}. Let *γ* = $v_{1}$. Consider the monophonic path P: $v_{k}\to v_{k-1}\to v_{k-3}\to v_{k-4}\to ,\cdots ,\to v_{3}\to v_{1}$ if k≡2(mod3). On placing $2^{k-(\lceil \frac{k}{3}\rceil +1)}$ - 1 pebbles on the path P, the destination is not reached. Therefore, the monophonic rubbling number of square of paths $\left(P_{k}^{2}\right)$ is $\mu_{r}(P_{k}^{2})$ ≥ $2^{k-(\lceil \frac{k}{3}\rceil +1)}$.

To prove the sufficiency condition, let D be any scattering of $2^{k-(\lceil \frac{k}{3}\rceil +1)}$ pebbles on the vertices of the square of paths $P_{k}^{2}$.

**Case 1:** Let *γ* be any end vertex. Let *γ* = $v_{k}$.

Consider the monophonic path $P_{1}$: $v_{1}\to v_{3}\to v_{5}\to ,\cdots ,\to v_{k-1}\to v_{k}$. The monophonic length from *γ* to any other vertex is atmost $k-(\lceil \frac{k}{3}\rceil +1$). On placing $2^{k-(\lceil \frac{k}{3}\rceil +1)}$ pebbles on the path $P_{1}$, the destination is reached. On placing most $2^{k-(\lceil \frac{k}{3}\rceil +1)}$ - 1 pebble on the path $P_{1}$ and placing one pebble on any one of the remaining vertices, by Theorem 6.1., the destination is reached. Hence, the destination is reached for all the scattering of pebbles on the vertices of the graph $P_{k}^{2}$. Similarly, we can prove for $v_{1}$.

**Case 2:** Let *γ* = $v_{d}$ where d = {2,3,5,⋯,k−1}.

The monophonic distance from $v_{d}$ to $v_{i}$ is atmost $k-(\lceil \frac{k}{3}\rceil +1+d)$ where d < i ≤ k. If $p(<P_{k}^{2}-\{v_{d},v_{d+1},v_{d+3},v_{d+4},\cdots ,v_{k-2},v_{k-1},v_{k}\}>)$ contains atleast $2^{k-(\lceil \frac{k}{3}\rceil +1+d)}$ + $\lceil \frac{k}{3}\rceil +d$ pebbles, the destination is reached by Case 1. Otherwise, if $<\{v_{1},v_{2},v_{3},\cdots ,v_{d}\}>$ has at least $2^{d-(\lceil \frac{d}{3}\rceil +1})+\lceil \frac{k}{3}\rceil$ + (k - d) pebbles, the destination is reached by Case 1 since the monophonic length from $v_{d}$ to $v_{j}$ is at most $\left(d-(\lceil \frac{d}{3}\rceil +1)\right)$, where 1 ≤ j < d. Therefore, the monophonic rubbling number of square of paths $P_{k}^{2}$ is $\mu_{r}(P_{k}^{2})$ = $2^{k-(\lceil \frac{k}{3}\rceil +1)}$. Hence the proof.

## 7 Comparative analysis table

**Table:** 

Article	Limitation	Our
		Results
Lourdusamy et al.		
(2022) [13]	*μ*(*C*_*k*_)=2^*k*−2^ + 1	*μ*_*r*_(*C*_*k*_) = 2^*k*−2^
Lourdusamy et al.		
(2022) [13]	*μ*(*K*_*k*_)=k	*μ*_*r*_(*K*_*k*_)=2
Lourdusamy et al.		
(2022) [13]	*μ*(*W*_*k*_)=2^*k*−2^ + 2	*μ*_*r*_(*W*_*k*_)=2^*k*−3^
Lourdusamy et al.		
(2022) [13]	*μ*(*S*_1,*k*_)=k + 2	*μ*_*r*_(*S*_1,*k*_)=4
Lourdusamy et al.		
(2022) [13]	*μ*(*F*_*k*_)=2^*k*−2^+1	*μ*_*r*_(*F*_*k*_)=2^*k*−2^
Lourdusamy et al.		
(2022) [11]	$\mu (P_{k}^{2})$=$2^{k-(\lceil \frac{k}{3}\rceil +1)}$	
	+ $\left(\lceil \frac{k}{3}\rceil \right)$	$\mu_{r}(P_{k}^{2})$=$2^{k-(\lceil \frac{k}{3}\rceil +1)}$

## 8 Limitations

Here we discuss the limitations of the obtained results in this article.

• The monophonic rubbling number primarily focuses on theoretical aspects rather than practical problems.
• To compute the monophonic rubbling number for any graph there is no well-known algorithm.
• The concept is computationally expensive as it is an NP-complete problem.

## 9 Conclusion

The monophonic rubbling number is a model which is applicable for transporting resources across the network. It finds applications in optimising resource allocation in communication networks, sensor networks and transportation logistics. We gave the motivation behind introducing the monophonic rubbling number concept into the literature. Here we computed the lower bound and upper bound of the monophonic rubbling number of the path graph, cycle graph, the complement of cycle graph, complete graph, wheel graph, k-star graph, complete bipartite graph, fan graph, ladder graph, middle graph of a path graph and square of paths. We compared results obtained using the monophonic pebbling number concept with our results. We conclude with the remarks that the monophonic rubbling number is the most advanced and useful technique in reducing the minimum number of pebbles compared with the classical techniques. The future direction of the article will be to focus on network-related graphs like probabilistic neural network, cellular neural network, tickysym spiking neural network mentioned in [20] and hexagonal cellular networks mentioned in [21], fuzzy soft graphs mentioned in [22], and convolutional neural networks mentioned in [23].

## CRediT authorship contribution statement

**K.C. Kavitha:** Methodology. **S. Jagatheswari:** Writing – review & editing, Supervision, Investigation.

## Declaration of Competing Interest

The authors declare that they have no known competing financial interests or personal relationships that could have appeared to influence the work reported in this paper.

## Data Availability

There was no data used for the research conducted in the article.
